# Ferumoxytol-enhanced MR imaging for differentiating intrapancreatic splenules from other tumors

**DOI:** 10.1007/s00261-020-02883-y

**Published:** 2020-12-30

**Authors:** M. R. Muehler, V. R. Rendell, L. L. Bergmann, E. R. Winslow, S. B. Reeder

**Affiliations:** 1 Department of Radiology, University of Wisconsin, Madison, WI USA; 2 Department of Radiology and Neuroradiology, University Greifswald, Greifswald, Germany; 3 Department of Surgery, University of Wisconsin, Madison, WI USA; 4 Department of Radiology, University of Texas Southwestern, Dallas, TX USA; 5 Medstar Georgetown Transplant Institute, Medstar Georgetown University Hospital, Washington, DC USA; 6 Department of Biomedical Engineering, University of Wisconsin, Madison, WI USA; 7 Department Medical Physics, University of Wisconsin, Madison, WI USA; 8 Department of Medicine, University of Wisconsin, Madison, WI USA; 9 Department of Emergency Medicine, University of Wisconsin, Madison, WI USA

**Keywords:** Pancreas, Ultra-small super paramagnetic iron oxides (USPIO), Neuroendocrine tumor, Splenule, Magnetic resonance imaging (MRI), Ferumoxytol

## Abstract

**Objectives:**

Ferumoxytol is an ultra-small superparamagnetic iron oxide (USPIO) agent that is taken up by splenic tissue. This study describes our initial institutional experience of ferumoxytol-enhanced MRI (feMRI) for differentiating intrapancreatic splenules (IPS) from other pancreatic lesions.

**Methods:**

In this retrospective study, patients with computed tomographic imaging that identified small enhancing lesions in the tail of the pancreas subsequently underwent feMRI for further characterization. The feMRI protocol included T2-weighted (T2w) imaging with and without fat suppression (FS), R2* mapping, diffusion-weighted imaging (DWI), and T1-weighted (T1w) imaging with FS, prior to contrast injection. Immediately after slow intravenous infusion with 3 mg/kg body weight ferumoxytol, T1w was repeated. Delayed imaging with all sequences were obtained 24–72 h after ferumoxytol administration.

**Results:**

Seven patients underwent feMRI. In two patients, the pancreatic lesions were presumed as pancreatic neuroendocrine tumor (PNET) from feMRI and in the remaining 5 IPS. One of the two patients with PNET was symptomatic for NET. In another symptomatic patient with pathologically proven duodenal NET and suspected PNET, the pancreatic lesion was proven to be an IPS on feMRI. IPS demonstrated strong negative enhancement in feMRI on T2w and increased R2* values consistent with splenic tissue, while the presumed PNETs did not enhance. T2w FS was helpful on the pre-contrast images to identify IPS, while R2* did on post-contrast images. Neither DWI nor T1w contributed to differentiating PNETs from IPS.

**Conclusions:**

This study demonstrates the potential utility of feMRI as a helpful adjunct diagnostic tool for differentiating IPS from other pancreatic lesions. Further studies in larger patient cohorts are needed.

## Introduction

Splenules (also known as splenunculus, accessory or supernumerary spleens) are ectopic splenic tissue [1, 2]. They are considered a developmental normal variant and have a high prevalence, with up to 10–30% of individuals having between one to six splenules [1–7]. The radiological and pathological literature report that approximately 75% of splenules are located in the splenic hilum, 20% near or in the pancreatic tail, and 5% in other regions. Cases of splenules found in the pelvis, groin, gonads, kidneys, adrenal glands, gastrosplenic ligament, liver, gastric wall, intestinal wall, omentum, and mesentery, as well lungs have been reported [2, 4, 8–15].

Splenules are frequently found as incidental findings during imaging and may be difficult to differentiate from other lesions [16]. In particular, intrapancreatic splenules (IPS) can mimic pancreatic neuroendocrine tumors (PNETs) [17, 18]. Both IPS and PNETs are often well circumscribed, enhancing lesions when imaged after bolus injection of extracellular contrast agents using computed tomography (CT) or magnetic resonance imaging (MRI) [6, 16, 17, 19–26]. While IPS are benign and require no intervention, PNETs are an uncommon and heterogeneous group of endocrine tumors that have malignant potential [27]. While some PNETs are functional, i.e., present with symptoms related to inappropriate excretion of various active hormones (e.g., gastrin, insulin, etc.), the majority are asymptomatic and non-functional. Moreover, non-functional PNETs have a worse prognosis with overall 5-year survival rate of 30%, compared to 97% with functional PNETs [28]. Preoperative biopsy of pancreatic tumors has not been shown to rule out malignancy with adequate reliability [28, 29]. Therefore, the need for a reliable imaging modality to differentiate IPS and PNETs is important for prognostic and therapeutic purposes.

When a splenule is suspected, the current imaging method of choice is 99mTc labeled heat-damaged red blood cell scintigraphy (99mTc-HDRBC) [5, 18, 30]. Although highly specific, scintigraphy is limited by low spatial resolution compared to CT and MRI, leading to low accuracy for evaluating lesions smaller than 20 mm. Unfortunately, the majority of intrapancreatic splenules, are smaller than 20 mm ranging typically from 10 to 15 mm in diameter [1, 3, 8, 18]*.*

An alternative approach of using ultra-small superparamagnetic iron oxide particles (USPIO) for imaging the mononuclear phagocyte system (MPS, formerly known as the reticuloendothelial system, or RES) has been proposed and investigated [11, 31–36]. Like 99mTc-HDBRC, the use of USPIO is based on the phagocytosis of particles by the MPS [37–39]. This approach has not been adopted clinically due to the lack of approval of USPIO contrast agents and withdrawal of some agents from the market [39–41].

In 2009, the US Food and Drug Administration (FDA) approved ferumoxytol (Feraheme, AMAG Pharmaceuticals, Waltham, MA, USA) to treat iron deficiency anemia in adults with chronic kidney disease (CKD) [42, 43]. Importantly, this agent has favorable MR imaging properties, and there has been growing off-label use of ferumoxytol by clinicians and researchers as an MR contrast agent for both positive contrast T1-weighted (T1w) and negative contrast T2-weighted (T2w) imaging [42]. Organs of the MPS, including the spleen, demonstrate strong negative enhancement in T2w MRI 24–72 h after the administration of ferumoxytol, while pancreatic tissue does not [42–44]. Based on the imaging properties of ferumoxytol, our institution has adopted the use of ferumoxytol for the characterization of small pancreatic tail masses.

The purpose of this retrospective study was to describe our institution’s experience with ferumoxytol-enhanced MRI (feMRI) for differentiating IPS from other types of pancreatic lesions.

## Methods

Our institution began formally offering feMRI in October 2014 after implementing a routine clinical protocol. This IRB-approved, retrospective study reviewed all feMRI studies performed for characterization of enhancing lesions in the tail of the pancreas. Patient demographics and relevant clinical information were retrieved from the electronic medical record.

### Imaging protocol for feMRI

MRI was performed on various 1.5 and 3.0 T clinical MRI systems using 8–32 channel phased array torso coils (GE Healthcare, Waukesha, WI) (Table 1). For each patient, an initial MRI (MR#1) and a delayed MRI (MR#2), obtained 24–72 h after intravenous (IV) administration of ferumoxytol were always performed on the same MRI system.

**Table 1:** List of patients in chronological order with clinical information and radiological findings of their intrapancreatic lesions

Pt	Age (years)	Sex	Clinical information					Radiological findings ferumoxytol study					Diagnosis
			Indication for imaging	Laboratory findings	Symptoms	Other workup	Pathology results	Size (mm)	MRI #1 (dynamic MRI) SI IPL T2 w/o FS		MRI #2 (delayed MRI) SI IPL T2 w/o FS		
									vs. pancreas	vs. spleen	vs. pancreas	vs. splIen	
1	59	M	Kidney donor	None	None	CT for kidney donor	None	6	Iso	Iso	Hypo	Iso	Splenule
2	52	M	Surveilling liver cyst	Chromogranin A 111 ng/mL*	None	two negative follow-up studies	EUS-FNA undetermined	17	Iso	Iso	Hypo	Iso	Splenule
3	67	M	Left rib pain	Synaptophysin and chromogranin A normal	None	biliary and pancreatic duct stent placed	None	16	Slightly hyper	Hyper	Hypo	Iso	Splenule
*4*	*73*	*M*	*Incarcerated hernia*	Chromogranin A 1576 ng/ml*	*None*	*None*	*None*	*15*	*Hypo*	*Hypo*	*Hypo*	*Slightly hyper*	*PNET*
5	41	F	Thoracic outlet syndrome	None	None	CT of the thorax	None	5	Iso	Iso	Hypo	Iso	Splenule
*6*	*77*	*M*	*Suspicion of insulinoma*	Chromogranin A 52 mg/ml* CA 19–9 5 U/mL#	*Recurrent hypoglycemic episodes*	*CT and several clinical test confirming suspicion*	*PNET with positive lumph nodes (pT1N1)*	*13*	*Hypo*	*Hypo*	*Hypo*	*Iso*	*PNET*
7	57	M	Epigastral pain, nausea and vomiting	Chromogranin A 914 mg/ml* Glucagon 359 ng/L^¤^	Poorly controlled diabetes	CT and DOTATE PET/CT confirming the pancreatic lesion suspicous of NET	Pancreatic lesion suspicious of NET after EUS-FN	17	Iso	Iso	Hypo	Iso	Splenule

Patients with diagnosis of PNET are highlighted in italics. (*normal 0–95 ng/mL, #normal: 0–37 U/mL;.¤normal: ≤ 208 ng/L)

The feMRI was performed as follows: Pre-contrast MR #1 axial and coronal 2D T2w single shot fast spin echo (T2-SSFSE, TR 700–2600 ms, TE 80–250 ms, SL 2–4 mm slice), 2D axial T2-weighted fast spin echo with fat suppression (FS) (T2w-FSE FS, TR 2.4–15 s, TE 90–98 ms, 4–5 mm slice), axial 3D T1w spoiled gradient echo with FS (LAVA-Flex, TR 3.6–4 ms, TE 1.7–1.9 ms, 12–15° flip angle, 3–5 mm slice), 3D axial multi-echo chemical shift encoded MRI (CSE-MRI) (IDEAL IQ, TR 6.6–13.8 s, TE 1.7–1.9 ms, 3–7° flip, 3–7 mm slice, 6–8 echoes per TR) for fat-corrected complex R2* mapping, and 2D diffusion-weighted imaging (DWI, TR 13.3–21 s, TE 51–63 ms, 4–5 mm slice, *b* = 50 and 500 s/mm^2^). The number of slices for each of the above acquisitions were adjusted to cover the relevant anatomy of interest, based on prior imaging. All acquisitions were performed in a breath-hold (20 s or less), except T2w-FSE FS and DWI, which were performed using respiratory triggering.

A slow intravenous infusion of 3 mg/kg body weight ferumoxytol diluted fivefold with normal saline was administered over at least 15 min outside of the MRI system [45]. The patients remained under observation for side effects starting 5 min prior to and 30 min after infusion, in accordance with the FDA Drug Safety Communication [45, 46]. After injection, T1w imaging was repeated.

Delayed MR #2 was performed 24–72 h after ferumoxytol administration using the same protocol as MR #1. In all cases, MR #2 was performed on the same MRI system as MR #1.

### Image analysis

Regions of interest (ROIs) were measured in corresponding areas of axial T2w-SSFSE in the liver, spleen, pancreas, intrapancreatic lesion (SI_tissue_), and fat (SI_fat_) both in the MR #1 as well MR #2 using McKesson PACS (McKesson Radiology Station, Version 12.3, Richmond, BC, Canada). Circular ROIs were drawn as large as possible while conforming to anatomic boundaries and avoiding large vessels, bile ducts, etc.

To compensate for signal inhomogeneities caused by sensitivity issues of the coils, every SI_fat_ was measured as close to the SI_tissue_ in the adjacent retroperitoneal adipose tissue as possible, and normalized signal intensity SI_norm_ was calculated as follows:

$${\mathrm{SI}}_{\mathrm{norm}}=\frac{{\mathrm{SI}}_{\mathrm{tissue}}}{{\mathrm{SI}}_{\mathrm{fat}}}$$

Qualitative assessment of the relative signal intensity of the intrapancreatic lesion to the pancreas and spleen both before and after contrast administration was performed for the T1w and diffusion-weighted images.

Due to the low R2* values and consequently low contrast on the pre-contrast R2* mapping sequence it was difficult to reliably delineate the upper abdominal organs, and the IPS in particular. For this reason, only qualitative assessment of iron uptake before and after ferumoxytol administration was performed relative to the spleen.

## Results

Between October 2014 and January 2020, 7 patients (6 men and 1 woman, age range 41–77 years) underwent feMRI as part of their clinical workup for an enhancing intrapancreatic tail lesion observed on prior imaging. No side effects due to the ferumoxytol administration were experienced by any patients in this study.

### Clinical presentation

The clinical presentation of the 7 patients are summarized in Table 1. In 5 patients, the indication for feMRI was based on an incidental, hypervascular intrapancreatic tail lesion (average size 11 mm, size range 5–17 mm) of unknown etiology identified on a prior imaging study as follows: MRI to exclude malignancy in a possible kidney donor (*n* = 1); chest CT for thoracic outlet syndrome (*n* = 1); and abdominal CT (*n* = 4) for cystic liver lesions, left rib pain, and a right inguinal hernia. In one patient (pt. 6) clinical symptoms had raised suspicion of an insulinoma for which imaging was obtained. In another patient (pt. 7) a CT was obtained due to epigastric pain and a complex clinical picture concerning for glucagonoma.

Of the 5 asymptomatic patients, three patients (pts. 1, 3, 5) had no notable laboratory derangements, and imaging findings of the ferumoxytol MRI supported the diagnosis of IPS.

One patient (pt.2) had a mildly elevated chromogranin A (CgA) level of 111 ng/mL (normal 0–95 ng/mL), but also suffered from asthma and took a serotonin specific reuptake inhibitor, which are both factors that can lead to an elevation of CgA [47, 48]. An attempt to clarify the clinical picture with an endoscopic ultrasound and fine needle aspiration (EUS-FNA) of a pancreatic tail lesion yielded indeterminate results. In this case, the imaging findings of the feMRI strongly supported the diagnosis of an IPS.

A second asymptomatic patient (pt. 4) had a notably elevated CgA level of 1576 ng/ml demonstrated during the workup of an incidental lesion. The feMRI supported the diagnosis of a PNET. Due of the relatively small lesion size of 12 mm as well its stability in size in comparison to a CT obtained one year prior, a watchful waiting strategy was chosen. A follow-up CT 7 months after our feMRI did not demonstrate any lesion growth or any metastatic disease. Continued surveillance is planned.

One symptomatic patient (pt. 6) was admitted to our institution for altered mental status found to be the result of severe hypoglycemia. His workup included fasting insulin check during which he became symptomatically hypoglycemic within 6 h of fasting and was found to have an insulin level of 24 µIU/mL (normal 3–25 µIU/mL), and a proinsulin level of 10.6 µIU/mL (normal ≤ 8.0 µIU/mL). His clinical history revealed up to 15 recurrent hypoglycemic episodes over the prior year with visual changes, including blurry vision/diplopia and later dizziness, confusion, and aphasia. His labs revealed normal CgA (52 mg/ml) and cancer antigen (CA) 19–9 (5 U/mL, normal 0–37 U/mL) levels. Abdominal CT showed a well-delineated, contrast-enhancing, hyperdense, 13 mm lesion in the portal-venous phase. Based on this clinical presentation and the CT imaging findings, a functional PNET (insulinoma) was suspected [49]. The feMRI supported this diagnosis. The patient underwent distal pancreatectomy and splenectomy, and pathologic analysis of the lesion revealed a well-differentiated neuroendocrine tumor, Grade 1 (Ki-67 2.5%), measuring 15 mm, with lymphovascular invasion and metastasis to one of twenty-five lymph nodes (1/25) (pT1N1).

In another patient (pt. 7), an abdominal CT demonstrated a 17 mm enhancing lesion in the pancreatic tail, which was noted to be stable in size from previous imaging in 2013, and a newly detected enhancing 15 mm duodenal lesion. The patient’s family history was notable for a pulmonary neuroendocrine tumor. The patient had brittle diabetes and was found to have an elevated glucagon level of 359 ng/L (normal 0–95 ng/mL), which was concerning for the presence of a glucagonoma. The CgA level was also elevated to 915 ng/mL, although the patient was taking a daily proton pump inhibitor, which is known to elevate CgA levels. EUS-FNA of the duodenal lesion was performed, and pathology demonstrated a low- grade neuroendocrine tumor (KI-67 < 3%). Given the presence of the duodenal lesion the pancreatic tail lesion was suspicious of NET. 68 Ga-DOTA-TATE PET/CT showed intense focal uptake for both the duodenal and pancreatic tail lesion. There was uncertainty in a PNET diagnosis of the pancreatic tail lesion because of the negative follow-up. On feMRI, the imaging findings of the pancreatic tail lesion were most consistent with IPS.

### Imaging findings on MRI

#### IPS characteristics on feMRI

In the five cases with a presumed diagnosis of IPS (pts. 1–3, 5,7) feMRI showed consistent imaging patterns. For all sequences and phase of contrast, all IPS followed identical imaging characteristics as the spleen. Specific behavior is described below.

**Fig. 1:**
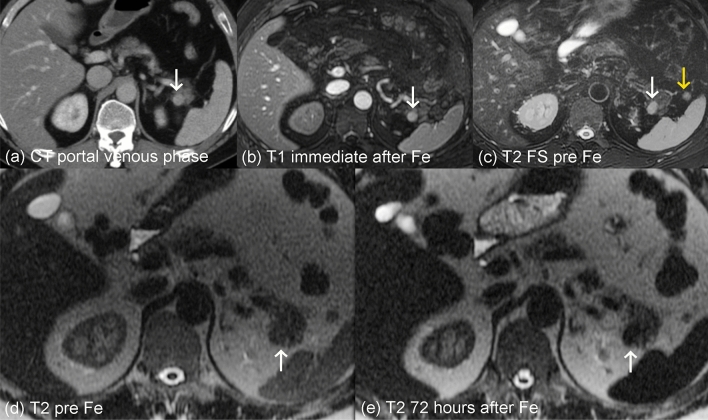
Typical imaging behavior of an intrapancreatic splenule on feMRI (pt. 2). CT shows enhancing lesion (white arrow, **a**), while T1w imaging shows strong enhancement immediately after administration of ferumoxytol (Fe) in an intravascular phase at MRI#1, similar to the spleen (**b**). On T2w imaging, the lesion is isointense before contrast (**c**, **d**) and demonstrates decreased signal, also similar to the spleen, 72 h after the administration of ferumoxytol (**e**). Note an additional splenule in the hilus of the spleen (yellow arrow)

**Fig. 2:**
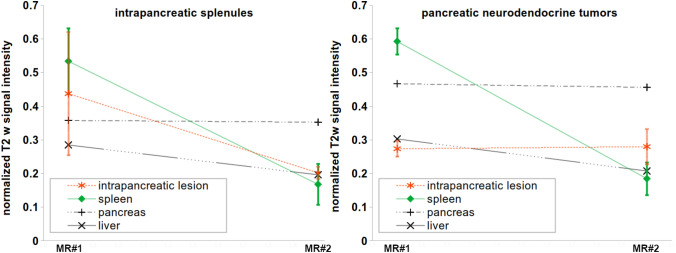
Normalized signal intensities on T2w imaging without fat saturation can be used to help distinguish splenules from PNET using feMRI. The liver, spleen, and splenules all demonstrate a marked decrease in T2w signal intensity ratios after the administration of ferumoxytol, whereas the pancreas and two observed PNETs show no change in signal intensity ratios after ferumoxytol. The error bars on the measurements for spleen and intrapancreatic lesions represent minimal and maximal values with each region of interest

**Fig. 3:**
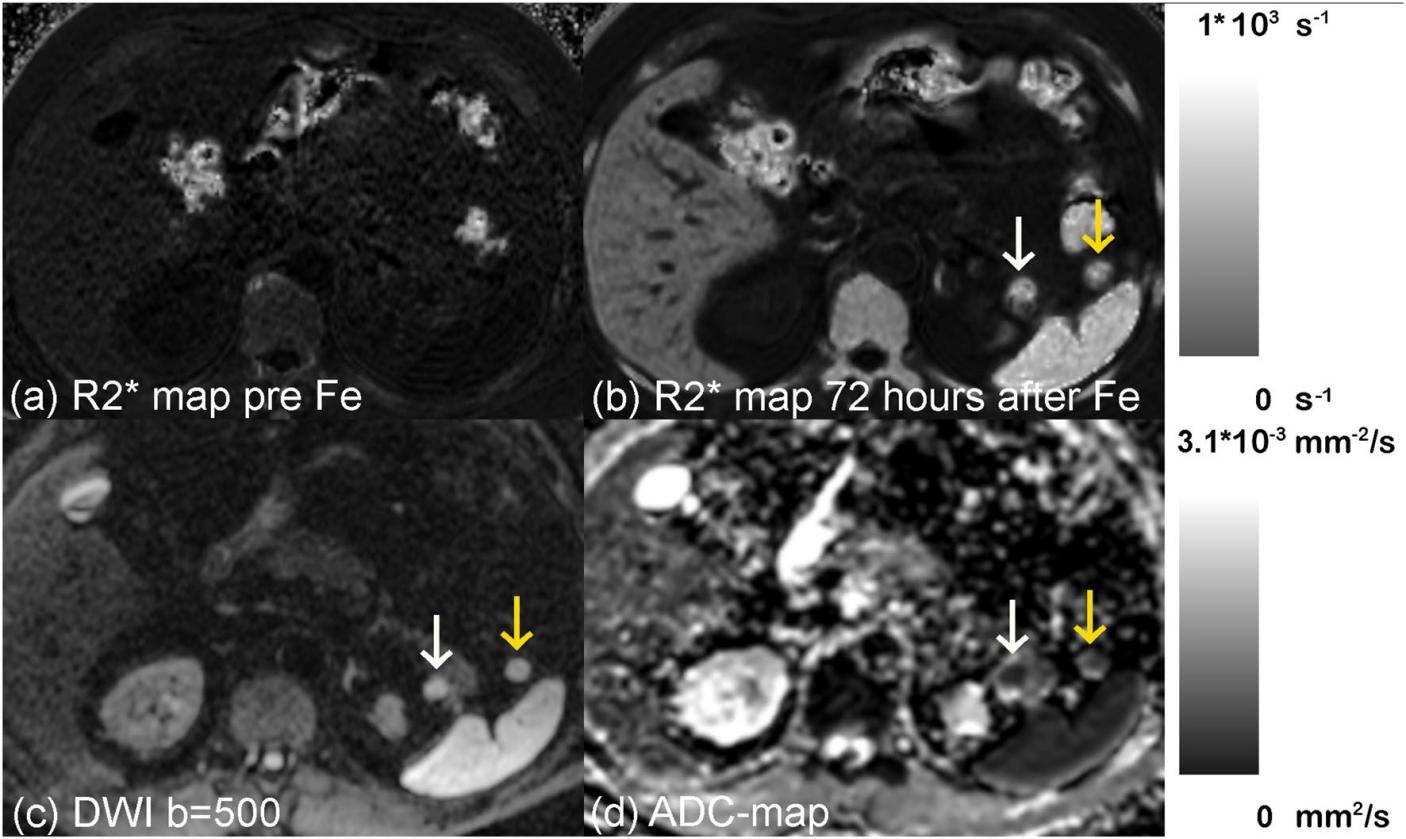
R2* mapping and DWI in a presumed splenule (pt. 2). Pre-contrast R2* maps demonstrate low R2* in the upper abdominal organs (**a**). After administration of ferumoxytol, the USPIO particles are phagocytosed by the MPS. 72 h after ferumoxytol administration, the intrapancreatic splenule (white arrow), a splenule in the hilus of the spleen (yellow arrow), the liver, and the spleen all show elevated R2* values (**b**). With DWI, the splenules have very similar signal intensity to the spleen on b = 500 s/mm2 (**c**) and similar ADC values as the spleen before ferumoxytol administration (**d**)

On T2w-SSFSE in the pre-contrast MRI, all IPS were isointense or slightly hyperintense to the pancreas and isointense to the spleen (Fig. 1). In the delayed MRI 24–72 h after the ferumoxytol administration, all IPS were hypointense to the pancreas and demonstrated a similar strong negative enhancement as the spleen (Fig. 2). Due to the uptake of USPIO, R2* maps showed a uniform and identical increased R2* in the IPS, spleen and liver in the delayed MRI compared to the pre-contrast MRI (Fig. 3).

All IPS behaved similarly to the spleen in pre-contrast T2w-FSE FS and DWI. On the pre-contrast T2w-FSE FS, the IPS were easily recognized as a well-delineated lesion that were homogeneously hyperintense compared to the pancreas. All IPS showed restricted diffusion in DWI similar to the spleen (Fig. 3). In the delayed MRI, all IPS were strongly hypointense relative to the pancreas with T2w-FSE FS. On the delayed MRI, the lesions were not visible on DWI, due to the T2 shortening from ferumoxytol.

On pre-contrast T1w-SGRE FS, the IPS appeared isointense or moderately hypointense to the pancreas. Immediately after the administration of ferumoxytol, the IPS appeared hyperintense, while in the delayed MRI (MR#2), they appeared isointense to the spleen. Of note, one patient (pt. 1) received gadobenate dimeglumine and was subsequently administered ferumoxytol 24 h prior to obtaining delayed imaging. This case was not included into the qualitative post-contrast T1 analysis.

The enhancement pattern of all five presumed IPS were consistent with a diagnosis of an intrapancreatic splenule as described in the literature [11, 35, 38, 39]. In 2 cases (pts. 2 and 5), additional accessory spleens measuring 6 and 15 mm were found in the hilum of the spleen (Fig. 1).

#### PNET characteristics on feMRI

**Fig. 4:**
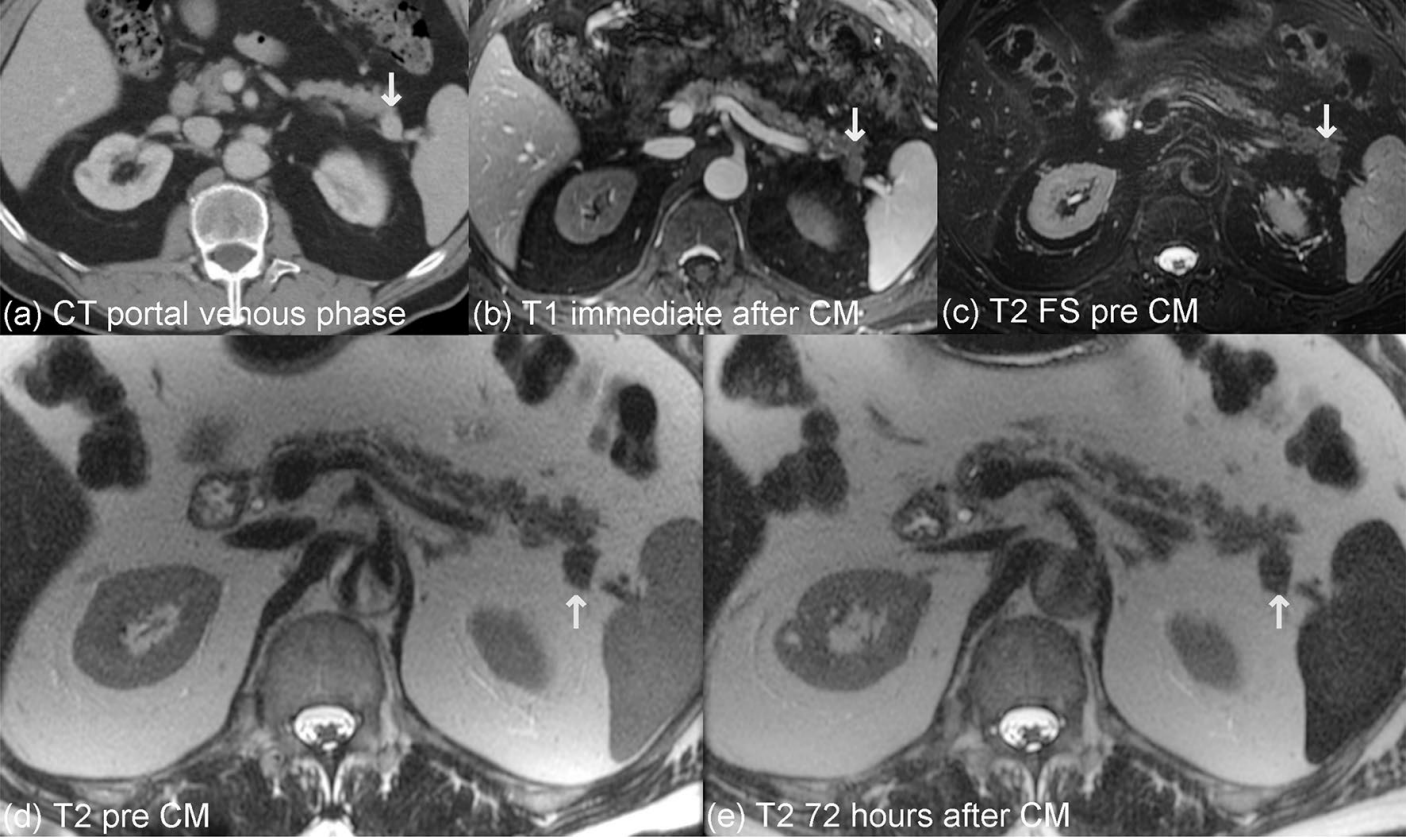
Imaging behavior of a pathologically proven PNET with feMRI (pt. 6). CT demonstrates an enhancing lesion (white arrow, **a**), which has relatively poor enhancement on T1w imaging immediately after administration of ferumoxytol in an intravascular phase at MRI #1, unlike the spleen (**b**). The PNET is hypointense to pancreas and spleen on T2w imaging (**c**, **d**) prior to contrast. 3 days after ferumoxytol administration, there is no change in the T2w signal of the lesion, unlike the liver and spleen which become relatively hypointense

In the two cases of presumed (pt. 4) or confirmed (pt. 6) diagnosis of a PNET, the contrast characteristics of these pancreatic lesions differed substantially from the IPS cases. With T2w-SSFSE on the pre-contrast MRI the PNETs were hypointense to the pancreas and spleen (Fig. 4). While the signal intensity (SI) of the liver and spleen in delayed MRI was markedly decreased on T2w-SSFSE, the PNETs remained unaltered (Fig. 2). Consequently, the PNETs appeared slightly hyper- or isointense relative to the spleen and had stable hypointensity relative to the pancreas (Fig. 4). R2* maps demonstrated increasing values in the liver and spleen on the delayed MRI in comparison to the pre-contrast MRI, but this pattern was not observed for the two PNETs.

On T2w-FSE FS the two PNETs were isointense to the pancreas in the immediate post-contrast and delayed MRI without any signal intensity change following ferumoxytol administration. Interestingly, the behavior in the DWI of the two PNETs was different. In one case (pt. 4) the PNET showed a similar restricted diffusion as the spleen; in the other (pt. 6) the PNET did not.

In the pre-contrast T1w-SGRE FS the lesions appeared isointense to the pancreas, and after ferumoxytol administration, no enhancement of either lesion was observed immediately after the administration of ferumoxytol.

The ferumoxytol contrast enhancement of the two PNETs differed significantly from the contrast behavior for IPS described in the literature [11, 35, 38, 39]. Consequently, the most probable diagnosis was thought to be PNET, confirmed histologically in one lesion.

## Discussion

In this work we have described our experience with feMRI for characterizing small pancreatic tail lesions, which was found to be helpful in distinguishing IPS from PNETs. All IPS cases demonstrated a consistent imaging pattern on feMRI that corresponded to the expected pharmacokinetics of ferumoxytol in the MPS, unlike lesions suspected to be PNETs.

The characterization of intrapancreatic tail lesions either IPS or PNET is essential for treatment planning. The primary goal is to identify all PNETs and avoid unnecessary surgical resection of IPS [24]. Clinical symptoms are uncommon with PNETs, and biomarkers and even biopsies are often unreliable [16, 47, 48, 50, 51]. Since PNETs can be very slow growing, follow-up imaging can also be of limited value to rule out potential malignancy [28]. Current CT and MRI cross-sectional imaging approaches using extracellular contrast agents do not allow reliable differentiation of these lesions [11, 22].

The current most specific imaging modality to differentiate IPS from PNETs, 99mTc-HDRBC, offers limited spatial resolution for lesions smaller than 20 mm and requires exposure to ionizing radiation [5, 18, 52]. 68 Ga-labeled somatostatin analogs (DOTA-TOC, DOTA-TATE, DOTA-NOC) for Positron Emission Tomography/Computed Tomography (PET/CT) has emerged as a reliable imaging modality for well-differentiated neuroendocrine tumors [53–55]. However, since splenic tissue also demonstrates high uptake of somatostatin analogs, this modality does not enable the differentiation of IPS from PNETs [56, 57]. Our clinical implementation of feMRI resulted in reliable distinction between IPS and PNETs. Additionally, all lesions we examined using feMRI were < 20 mm and were identified easily with the high spatial resolution of MRI.

As expected physiologically, all IPS demonstrated negative enhancement on delayed T2-weighted imaging after ferumoxytol administration in a manner consistent with splenic tissue. In contrast, the PNET cases did not demonstrate negative enhancement or follow the behavior of the spleen. Additionally, the liver and spleen demonstrated increasing R2* values after the administration of ferumoxytol, indicating the presence of iron uptake in the MPS. This corresponds with the known pharmacokinetics of USPIO by the MPS [11, 35, 36, 38, 39, 58]. The two PNET cases in our study showed variable behavior: one showed restrictive diffusion and one did not. This may be explained by variable tumor biology that can occur at different tumor stages [59, 60]. Thus, DWI alone may not enable the reliable differentiation of IPS from PNET, although further studies in larger numbers of patients would be needed to confirm this speculation [61–63].

Additionally, dynamic T1w imaging following ferumoxytol administration demonstrated strong positive enhancement for IPS, while PNETs did not show similar positive enhancement. Factors that may contribute to this phenomenon include varying microvascular density of the tumors, the long blood half-life of ferumoxytol (14–21 h), and the specific T1w sequence used [43, 64, 65].

In clinical scenarios where there is a need to characterize a pancreatic lesion suspicious for PNET vs IPS, we recommend a feMRI protocol comprising T2w without and with FS, R2* mapping before and 72 h after the administration of ferumoxytol.T2w-SSFSE without FS is helpful to confirm negative contrast enhancement of the lesion as well as give valuable anatomical information. The T2w-FSE with FS can help find all accessory spleens on the pre-contrast images. The use of delayed phase T2w imaging only, may prove inadequate because PNETs can appear hypointense on T2-weighted imaging prior to the administration of ferumoxytol, as observed in our experience and in previous work [66]. We found quantitative R2* mapping helpful because it facilitates the detection of splenules on delayed MRI. Quantitative R2* mapping on pre-contrast MRI is also helpful in the identification of preexisting iron overload which could limit the utility of feMRI. Given that ferumoxytol has a long blood pool phase, waiting for 24 h after the infusion to obtain delayed imaging, has proven sufficient in this study to identify negative enhancement [44]. In contrast, with 99mTC-HDRBC imaging can be performed as early as 30 to 60 min after the tracer injection [5, 34, 37, 42, 43, 58, 67–69].

Ferumoxytol also has a very good safety profile for both adults and pediatric patients with side effects demonstrated in 10–15% (most common: nausea, dizziness, and diarrhea) and severe side effects in up to 1%, similar to known rates with use of ionic iodinated contrast agents [42, 43, 45, 70, 71].

Ferumoxytol is generally more costly than gadolinium agents, but the price for the MRI scan at all is more expensive [45].

Our study had several limitations. First, it is retrospective in nature. Although this study is, to the best of our knowledge, the largest series reported in the literature, it includes a relatively small number of patients. Our study included all patients in our clinical experience over 5 years, reflecting the overall rarity of this clinical situation. Additionally, we do not have pathological correlation for all cases, which is due to our protocol being implemented clinically. Biopsies of small pancreatic tail lesions are known to be unreliable and are, therefore, infrequently obtained, and surgery was appropriately not performed for those cases with presumed IPS. While one PNET was removed surgically, the clinical team elected to follow a watchful waiting strategy for the second small PNET based on the small size, patient preference, and the fact that many PNETs never undergo malignant transformation. Lastly, we did not have direct comparison of feMRI with 99mTC HDRBC, since the lesions included in this study were considered too small for 99mTC-HDRBC.

In conclusion, we have demonstrated the feasibility of feMRI for the characterization of small pancreatic tail lesions in a clinical case series. With high spatial resolution and no ionizing radiation, ferumoxytol-enhanced MRI can be a valuable adjunct to, or a substitute for, scintigraphy and somatostatin analogs PET/CT and may satisfy an important unmet clinical need. Further evaluation of feMRI in larger cohorts of patients is needed to confirm our initial experience.
